# How Organisational Dynamics Impact Decision Latitude, Social Support, Self‐Identity Through Work and Job Insecurity for Nurse Practitioners

**DOI:** 10.1111/jan.16842

**Published:** 2025-02-24

**Authors:** Isabella Wright, Anja Vorster, Eamon Merrick

**Affiliations:** ^1^ School of Clinical Sciences Auckland University of Technology Auckland New Zealand; ^2^ Faculty of Health and Environmental Sciences Auckland University of Technology Auckland New Zealand; ^3^ Faculty of Health University of Technology Sydney Sydney New South Wales Australia; ^4^ Nursing and Midwifery Directorate Northern Sydney Local Health District Sydney New South Wales Australia

**Keywords:** nurse practitioner, nurse's roles, organisational behaviour, psychosocial factors, work environment

## Abstract

**Aim:**

To identify whether nurse practitioners (NPs) in New Zealand (NZ) have the organisational opportunities to make decisions related to performing their role.

**Design:**

A cross‐sectional study of self‐reported decision‐making, social support, psychosocial demands and identification with role in a representative population of NPs employed in a range of practice settings in NZ.

**Methods:**

This study utilised the internationally validated Job Content Questionnaire. Reliability and construct validity were assessed using co‐efficient α and confirmatory factor analysis. Linear regression analyses were conducted to understand the strength and direction of the relationships between the constructs.

**Results:**

All scales demonstrated acceptable levels of internal reliability. Factor analysis supported a five‐factor model, with *decision latitude, psychological job demands, co‐worker support, supervisor support* and *job insecurity* as the main factors fitting the theoretical model. Regression models suggested that NPs (*n* = 169) have more control over their decision‐making when supported by their colleagues rather than supervisors. NPs perceive improved relationships with healthcare consumers if they feel an increase in support from their colleagues; this relationship is mediated by the freedom to make decisions. NPs in rural settings had more job security when they felt valued and appreciated at work.

**Conclusion:**

The presence of collegial support significantly influences the freedom and autonomy of NPs in making decisions. Workforce policy, the organisation of practice and vocational training may be effective ways of helping NPs expand access to healthcare services.

**Implications for the Profession and Patient Care:**

Collegial and supervisory support are critical for NPs to work to their full scope. A funded, first‐year‐in‐practice vocational training program designed to support role transition, foster collegial support and build a community of practice for newly qualified NPs.

**Impact:**

For the first time, nurse practitioner decision‐making and autonomy determinants have been described in NZ. These findings should be considered within the context of international evidence and in global nursing workforce policies that seek to create opportunities for NPs to work to the limit of their scope.

**Reporting Method:**

The authors have adhered to relevant EQUATOR guidelines—STROBE checklist.

**Patient or Public Contribution:**

No patient or public contribution.

**Summary**

- What does this paper contribute to the wider global clinical community?
  ○ In New Zealand, collegial support is the most important determinant of nurse practitioner decision‐making.
  ○ A supportive work environment enables nurse practitioners (NPs) to exert greater control over their work and fosters meaningful patient relationships.
  ○ Rural NPs who perceive themselves as valued and appreciated experience enhanced job security.

## 1 Introduction

Nurse practitioners (NPs) are frequently presented as a workforce solution to expand access to healthcare services (Carryer and Adams 2017; Ploeg et al. 2013). Despite having a comparatively small population, New Zealand (NZ) faces significant barriers to accessing healthcare services, particularly in rural and underserved areas. New Zealand's healthcare system serves a population of 5.2 million people, is funded primarily by public sources and offers universal health coverage. Te Whatu Ora (Health New Zealand) oversees and operates all public hospitals and public health services. However, disparities in the healthcare system persist, particularly in terms of affordability, utilisation and quality of care. These inequities disproportionately affect the population living in deprived urban and remote rural communities, with Māori and Pacific Peoples experiencing the most significant disparities (Ministry of Health 2019). Other factors, such as low socioeconomic status, limited access to health services, and inequities in care quality, exacerbate these disparities, contributing to higher rates of poor health outcomes. These include long‐term conditions, cancer, oral health issues, smoking prevalence, obesity, substance misuse, suicide and family violence (Ministry of Health 2023).

Becoming a NP in NZ requires at least 4 years of experience as a registered nurse in a specific practice area and completing a clinical master's degree, including an approved prescribing practicum (NCNZ 2019). NPs are authorised to prescribe medications and independently assess, diagnose and manage patients within their specialised practice areas (Pirret 2008). While NPs can practice autonomously, most choose to work collaboratively within healthcare organisations alongside other health professionals. Despite the growing recognition of NPs potential to transform healthcare delivery, limited research exists on the workplace structures and organisational practices that enable them to maximise their contributions. For NPs to effectively exercise their autonomy in decision‐making and apply their advanced clinical skills, supportive environments are essential (Arendts et al. 2018; Forbes et al. 2018; Hagan and Curtis Sr. 2018; Lowe et al. 2013).

## 2 Background

The nurse practitioner role was introduced in NZ in 2001 to improve healthcare access and address the inadequate health outcomes experienced by the Indigenous Māori population and other marginalised communities. The 2016 NZ Health Strategy highlighted the need for a more integrated healthcare system, focusing on multidisciplinary collaboration and wellness preservation. The strategy emphasised the importance of aligning primary, secondary and tertiary services to address the rising prevalence of long‐term complex conditions requiring person‐centred, responsive and supportive services. The prevalence of long‐term conditions in NZ has increased by 62% from 1981 to 2013 due to the increasing life expectancy of older people aged 65 years and over. As the older adult population is predicted to increase by 84% in 2026, approximately 20,000 more individuals will require long‐term care (Ministry of Health 2016; Statistics New Zealand 2015).

Collaboration among primary and community healthcare providers was crucial in improving the management of these long‐term conditions (Ministry of Health 2016). At this time, the nurse practitioner workforce was promoted as essential in this evolving healthcare landscape. In NZ, NPs diagnose, treat and manage various health conditions. Their focus on health education, promotion and disease prevention equips them to address the complex needs of patients with long‐term conditions. Through ongoing care provision, symptom monitoring, referrals and collaboration with other health professionals, NPs offer comprehensive support to patients, thus alleviating the burden on medical practitioners (Adams and Carryer 2019; Carryer and Adams 2017; Carryer and Yarwood 2015).

Since the introduction of the role, NPs have been pivotal in enhancing healthcare access, particularly for marginalised and rural populations (Kooienga and Carryer 2015; Xue and Intrator 2016). However, the growth of the NP workforce has been relatively slow, especially during the first 13 years following their introduction (NCNZ 2020). NPs in NZ face several challenges that have hindered their professional development and integration into the healthcare system. These challenges include limited recognition and understanding of their role among colleagues and managers, insufficient funding, the dominance of biomedical models of care, and tensions among healthcare professionals (Adams et al. 2022). This slow uptake persisted despite robust international evidence supporting the safety, effectiveness, and value of NPs in meeting the healthcare needs of consumers (Carryer and Adams 2017; Barker et al. 2018; Burgess and Purkis 2010; Gagan et al. 2014; Sangster‐Gormley et al. 2013).

The international literature identified several critical factors contributing to the successful integration and practice of NPs, including support from healthcare teams, collegial relationships and organisational support. These factors were associated with improved job satisfaction, greater clinical autonomy and increased visibility of NPs within healthcare systems (Alexander et al. 2023; Poghosyan et al. 2020; Whitehead et al. 2022). For these reasons, it is essential to understand how NPs experience the organisation and structure of their work. In particular, the relationship between decision‐making, social support and how NPs identify with their role. Understanding these dynamics could provide valuable insights into improving healthcare delivery and optimising patient outcomes within the NZ healthcare system.

## 3 The Study

### 3.1 Aim

To identify whether NPs in NZ have the organisational opportunities to make decisions related to performing their role.

#### 3.1.1 Research Questions

1. Does support from colleagues and/or supervisors influence NPs freedom to make decisions about practice?
2. Is there a relationship between social support and nurse practitioner‐patient (customer) relationships, and does the freedom to make decisions about practice influence this relationship?
3. Is the geographical location of practice associated with the freedom that NPs have in making decisions about practice?
4. Is there a relationship between work commitment and job insecurity for NPs, and if so, does the geographical location moderate this relationship?

### 3.2 Methods

A cross‐sectional design using the Job Content Questionnaire (JCQ) was used. The JCQ is an instrument that measures constructs derived from the Job Demand‐Control Support (JDCS) model. The specific constructs assessed in this study include (1) *decision latitude*, (2) *social support*, (3) *psychological demands*, (4) *job insecurity*, (5) *customer relationships* and (6) *self‐identity through work* (Karasek et al. 1998).

### 3.3 Measurement

The JCQ has developed significantly since its inception in 1984 and was expanded with additional items in 2015 to enhance comprehensiveness. The questionnaire offers flexibility in its format to accommodate various research needs, as outlined in the JCQ user's guide (Karasek 2015). In this study, the JCQ was not modified as its statistical reliability, internal scale reliability and construct validity is well‐documented in prior research (Amin et al. 2015; Amost and Laschinger 2005; Baba et al. 2013; Dery et al. 2022; Merrick et al. 2012; Sasaki et al. 2020). Previous JCQ studies reported Cronbach's alpha (*α*) values for its constructs ranging from 0.74 to 0.91 (Navajas‐Romero et al. 2020), 0.71 to 0.76 (Dery et al. 2022) and 0.66 to 0.86 (Merrick et al. 2012).

The JCQ in this study comprised 31 items and contained a complete set of questions for assessing five scales. The scales included: (1) ‘*decision latitude*’ that measures an individual's ability to control tasks and work activities. It pertains to the methods and means chosen to perform a specific role. *Decision latitude* served as the primary dependent variable in this study, a composite construct comprised of two sub‐scales: *skill discretion* and *decision authority*. *Skill discretion* (six items) reflects an individual's ability to utilise specific job skills, while *decision authority* (three items) represents a person's autonomy in decision‐making, including control over timing and methods. (2) A scale of ‘psychological job demands’ that measures various aspects such as workload, constraints on task completion, and conflicting demands in the workplace. In this study, psychological job demands (five items) are referred to as the demands of performing a role. (3) A ‘*social support*’ scale measures opportunities for a person to be supported at work. *Social support* is divided into two composite sub‐scales: *co‐worker support* and *supervisory support*. *Co‐worker support* (four items) pertains to the amount of support the individual receives from the people they work with. *Supervisory support* (four items) denotes the level of support a person receives from their immediate supervisor or manager (Häusser et al. 2010). (4) The identity obtained from the NP role was assessed by the scale ‘*self‐identity through work*’ (six items), which explains the importance of a person's work being recognised by others, such as a customer, company or society. (5) The scale of ‘*job insecurity*’ (three items) is associated with work‐related psychological burdens, encompassing concerns about potential unemployment and restricted future career development opportunities (Zolnierczyk‐Zreda and Bedynska 2014). Each scale was measured using a four‐point Likert response format, ranging from 1 (strongly disagree) to 4 (strongly agree) (Karasek 2015). The demographic variables collected in this study included gender, age, ethnicity, current employment setting, clinical speciality area, geographical location of main practice (postcode) and number of years of practice as an NP.

### 3.4 Study Setting and Sampling

In September 2020, NPs in NZ were invited to participate in the online self‐administered questionnaire. Invitations to the survey were emailed to NPs affiliated with Nurse Practitioners New Zealand (the NPNZ), a professional association that is a part of the College of Nurses Aotearoa. The recruitment strategy spanned 1 month and involved the NPNZ sending a link to the study via Qualtrics^XM^ survey software. The inclusion criteria were NPs registered with the Nursing Council of New Zealand and in current clinical practice, NPs were the population of interest in this study.

### 3.5 Data Analysis

Scales were considered reliable if their *α* values fell within the acceptable levels of 0.61 and 0.90 (Sasaki et al. 2020; Van Poel et al. 2020). This range was used to assess the internal consistency of the measurement and its subscales. Using Spearman's rank correlation, inter‐correlations were calculated to examine bivariate relationships in the different sub‐scales. Confirmatory factor analysis was conducted to assess the construct validity of the JCQ within the present sample, following the methodology of previous studies (Sasaki et al. 2020; Zolnierczyk‐Zreda and Bedynska 2014). The confirmatory factor analysis results provided insights into the contribution of factors to the theoretical model. The model fit was evaluated based on the fit indices reported in prior JCQ research (Niedhammer et al. 2020; Orgambidez and Almeida 2020; Roelen et al. 2014), ensuring consistency across different indices to support a good‐fitting model.

Linear regression models were developed and tested to explore the relationships between constructs. First, simple regression was used to assess how *social support*, as the predictor, influences NPs' *decision latitude* (outcome). Then, multiple regression was used to evaluate the distinct effects of *co‐worker support* and *supervisory support* on decision latitude. A mediation model was tested to explore the role of *decision latitude* as a mediator between *social support* and *customer relationships* (as the outcome variables). Furthermore, the influence of *self‐identity through work* (predictor) on *job insecurity* (outcome) was examined. Whether geographical location moderated this relationship was tested by creating an interaction term between *self‐identity* and geographical location. The analyses included simple and multiple regression, mediation and moderation analysis to address the research questions. Bootstrap analysis was applied with 1000 iterations and 95% confidence intervals. Analyses were conducted using SPSS version 28 [including the PROCESS macro by Andrew F. Hayes (2022) and AMOS version 28.0].

### 3.6 Ethical Considerations

Approval for this research was sought and obtained from the Auckland University of Technology Ethics Committee (AUTEC# 20/194) on July 31, 2020.

## 4 Results

### 4.1 Characteristics of the Sample

During the recruitment period, 194 respondents completed the questionnaire, accounting for 55% of the total 350 NP workforce listed in the Nursing Council New Zealand's public register of nurses (NCNZ 2020). From the 194 questionnaires received, 25 respondents were excluded from the analysis as more than 80% of their responses were missing. A final sample of 169 (*n* = 169) NPs, representing 49% of the NP workforce in 2020, was obtained during the survey distribution.

Among the 169 respondents, 93.5% (*n* = 158) identified as female, while 6.5% (*n* = 11) identified as male. Most respondents were 50 to 59 (40.8%), followed by 40 to 49‐year‐olds (27.8%). Most respondents identified as European, accounting for 87% of the sample. Māori and part‐Māori represented 7.1%, Asian and part‐Asian constituted 4.1%, and Pacific Peoples comprised 0.6% of the sample.

Most respondents have been practising as NPs for 2–5 years (53.8%). This was followed by those with over 6 years of experience (31.4%) and those with less than 1 year of experience (14%). The highest percentage of respondents (46.2%) were employed by District Health Boards (public hospitals). This was closely followed by those working in primary healthcare (36.1%), followed by NPs who work in residential aged care settings (4.7%), and those working in rural community practices (2.4%). Primary healthcare was the most common area of speciality (29.6%). The primary practice location for most NPs was metropolitan regions, accounting for 79.3% of respondents, and 20.7% of respondents were practising in rural areas with populations of less than 10,000. Table 1 displays the demographics of the sample (*n* = 169).

**TABLE 1:** Demographics of the sample (*n* = 169).

	Count	Percent
Nurse practitioner	169	100%
Gender		
Male	11	6.5%
Female	158	93.5%
Age (in years)		
25–29	3	1.8%
30–39	22	13.0%
40–49	47	27.8%
50–59	69	4.8%
60+	28	16.6%
Ethnicity		
European	147	87%
Māori + part‐Māori	12	7.1%
Pacific peoples	1	0.6%
Asian + part‐Asian	7	4.1%
Other	2	1.2%
Current employment setting		
Public hospitals	78	46.2%
Primary health care	61	36.1%
Residential aged care	8	4.7%
Rural/community	4	2.4%
Other	18	1.7%
Current area of clinical specialty		
Acute/critical care	8	4.7%
Emergency	16	9.5%
Paediatrics	7	4.1%
Primary health care	50	29.6%
Coronary care/heart failure	4	2.4%
Older adult	14	8.3%
Adult	4	2.4%
Diabetes	5	3.0%
Chronic conditions	3	1.8%
Respiratory	2	1.2%
Renal	5	3.0%
Palliative care	7	4.1%
Sexual and women's health	4	2.4%
Mental health	8	4.7%
Other	32	18.9%
Length of time practising as an NP		
Less than 1 year	25	14.8%
2–5 years	91	53.8%
Longer than 6 years	53	31.4%
Demographic location of main practice		
Metropolitan regions	134	79.29%
Rural (population < 10,000)	35	20.71%

### 4.2 Validity and Reliability

The internal reliability for all scales was above the acceptable level of 0.61, with values ranging between 0.63 and 0.89. The *decision latitude* scale formed a reliable measure (*α* = 0.69) of the two sub‐scales, *skill discretion* (*α* = 0.52) and *decision authority* (*α* = 0.58). The review of item‐total correlations for the *skill discretion* sub‐scale indicated correlations below the acceptable level of 0.3. However, removing these items did not improve the co‐efficient *α* to an acceptable level above 0.61, leading to the decision to retain all the items.

Results of the Spearman's rank correlation showed that *decision latitude* (i.e., the combined measure of *skill discretion* and *decision authority*) was significantly, yet weakly, correlated with all the other constructs [Spearman's rho (*ρ*) ranging between 0.18 and 0.50], but not with psychological *job demands*. The latter did not correlate significantly with any of the constructs measured by the JCQ. *Social support* correlated significantly with *decision latitude* (*ρ* = 0.22, *p* < 0.01) and *decision authority* (*ρ* = 0.19, *p* < 0.05). The strongest correlation was between *decision latitude* and *self‐identity through work* (*ρ* = 0.50, *p* < 0.01).

### 4.3 Confirmatory Factor Analysis

Three models were tested in the analysis. Model One is the six‐factor theoretical model that includes *skill discretion, decision authority, psychological job demands, supervisor support, co‐worker support* and *job insecurity*. Model Two and Model Three propose more parsimonious models. Model Two is a five‐factor model combining skill discretion and decision authority into *decision latitude*. Model Three combines *co‐worker* and *supervisor support* into the *social support* factor, creating a four‐factor model.

Several fit statistics were calculated to assess the model's goodness‐of‐fit of the observed data to the theoretical JCQ model. These included the chi‐square, the comparative fit index (CFI), the Tucker Lewis index or non‐normed fit index (NNFI), the PCLOSE, and the root mean square error of approximation (RMSEA) for three tested models. The results show that the goodness‐of‐fit statistics of both Model Two and Model Three improved over Model One. The CFI and the TFI increased to 0.93 and 0.92, respectively, and the RMSEA remained at an acceptable level of 0.05 for Model Two. These results indicate that Model Two, which combined skill discretion and decision authority sub‐scales into a single *decision latitude* scale, is the best‐fitting model for the data in the present study. This suggests that *skill discretion* and *decision authority* might not be distinct factors in the present study but components of the broader *decision latitude* factor. Therefore, to enhance the measurement accuracy of the JCQ, *decision latitude* was treated as an integrated measure in the subsequent analyses of this study.

### 4.4 Regression Models

To address research question one: ‘Does support from colleagues and/or supervisors influence NPs freedom to make decisions about practice?’ a series of regression models were tested. First, a simple regression model was conducted with *decision latitude* as the outcome and *social support* as the predictor variable. The analysis (using bootstrap analysis with 1000 iterations and a 95% confidence interval) showed a significant relationship between *social support* and *decision latitude*, explaining 6% of the variance (*R* ^2^ = 0.06, *b* = 0.54, *t* = 3.22, *p* < 0.01, 95% CI [0.21, 0.87]). Next, a multiple regression analysis tested the distinct roles and influence of *co‐worker* and *supervisory support. Co‐worker support* had a significant positive effect on *decision latitude*, *b* = 1.54, *t* = 3.67, *p* < 0.001, 95% CI [0.71, 2.36], explaining 8.4% of the variance (adjusted *R* ^2^ = 0.08). *Supervisory support* did not show a significant effect, *b* = 0.28, *t* = 1.46, *p* = 0.15, 95% CI [−0.10, 0.66]. Adding *supervisory support* to the model only slightly increased the variance explained (Δ*R* ^2^ = 0.01). The results indicated that *co‐worker support* has a stronger association with *decision latitude* than *supervisory support*.

To address research question two (Does *social support* influence nurse practitioner‐patient (customer) relationships, and is this effect mediated by *decision latitude*?), an indirect effect was tested between *social support* and nurse practitioner‐patient relationships via *decision latitude*. The analysis found that *social support* does not have a direct effect on patient relationships (*b* = 0.02, *t* = 0.47, *p* = 0.64, 95% CI [−0.06, 0.09]). However, *social support* indirectly influences patient relationships through *decision latitude* (*b* = 0.045, 95% CI [0.014, 0.083]), suggesting that higher levels of *social support* improve patient relationships by increasing *decision latitude*.

An independent samples *t*‐test was conducted to test whether the geographical location of practice is associated with the decision‐making freedom of NPs. This test compared NPs in rural areas with those in metropolitan regions regarding their perceived *decision latitude*. The results showed no significant difference in *decision latitude* between the two groups, *t* (155) = 0.91, *p* = 0.38, CI [−1.77, 4.78], *d* = 0.17. NPs in metropolitan areas (*M* = 80.53, SD = 8.75) and those in rural areas (*M* = 79.03, SD = 9.01) reported similar levels of perceived *decision latitude*. These results suggest that geographical location does not affect the decision‐making freedom of NPs in this study.

To address the final research question (Is there a relationship between work commitment and *job insecurity* for NPs, and does geographical location moderate this relationship?), it was tested whether the relationship between *self‐identity through work* and *job insecurity* was moderated by geographical location (rural vs. metropolitan).

The interaction term between *self‐identity* and geographical location was significant, *b* = 0.23, *t* = 2.15, *p* < 0.05, 95% CI [0.44, 0.02]. The overall model explained 9.3% of the variance in *job insecurity* (*R* ^2^ = 0.09). For NPs in rural areas, higher *self‐identity through work* was associated with lower *job insecurity* (*b* = −0.32, *t* = −3.53, *p* < 0.01, 95% CI [−0.50, −0.14]). This relationship was not significant for NPs in metropolitan areas (*b* = −0.09, *t* = −1.63, *p* = 0.11, 95% CI [−0.20, 0.02]). These findings indicate that the negative relationship between *self‐identity* and *job insecurity* (i.e., more self‐identity is related to lower *job insecurity*) is significant only for NPs in rural areas. Table 2 presents the results of a simple, multiple, mediation and moderation regression.

**TABLE 2:** Results of simple, multiple, mediation and moderation regression.

Predictor variables	Outcome variables	Coefficient (*b*)	*t*	*p*	95% confidence intervals	*R* ^2^
Simple regression						
Social Support	Decision latitude	0.54	3.22	< 0.01	[0.21, 0.87]	0.06
Multiple regression						
Co‐worker support	Decision latitude	1.54	3.67	< 0.001	[0.71, 2.36]	0.08 (adjusted)
Supervisory support	Decision latitude	0.28	1.46	0.15	[−0.10, 0.66]	0.09 (adjusted)
Mediation						
Social support (indirect effect through decision latitude)	Customer relationships	0.045	—	< 0.001	[0.014, 0.083]	0.13
Social support (direct relationship with customer relationships)	Customer relationships	0.02	0.47	0.64	[−0.06, 0.09]	—
Moderation						
Self‐identity interaction with geographical location	Job insecurity	0.23	2.15	< 0.05	[0.44, 0.02]	0.09

## 5 Discussion

### 5.1 Organisational Opportunities

In this study, NPs reported a stronger sense of freedom to make decisions about their practice when they felt supported by their colleagues. This positive relationship found in the results might improve autonomy for NPs. It is important to note that the concept of NP autonomy is sometimes misunderstood among health professionals (Weiland 2015). Peacock and Hernandez (2020) defined NP autonomy as utilising professional knowledge and expert clinical skills, having prescribing authority, taking responsibility for their practice, and engaging in unrestricted collaboration with an interdisciplinary health team. Lockwood et al. (2022) also explained that NP clinical autonomy includes self‐determination, competence, relatedness, autonomy, and stepping into higher leadership and clinical responsibilities. Autonomy is critical for NPs to effectively fulfil their roles, particularly in primary healthcare, where there are health workforce shortages (Carryer and Adams 2017; Carryer and Yarwood 2015).

NPs in NZ are highly skilled health practitioners who work across healthcare settings with a broad scope of practice. To fully support advanced practitioner roles like NPs, employers, regulatory authorities and speciality groups must express their endorsement (Adams and Carryer 2019, 2021). NPs' clinical autonomy is strengthened when health policy and organisational stakeholders collaborate intrinsically. Conversely, disconnecting health policy and organisational culture can extrinsically diminish NPs clinical autonomy (Lockwood and Schober 2024). The College of Nurses Aotearoa NZ (2019) has recognised the significance of supporting the NP in workforce development to enhance equity and access to competent and safe healthcare. The finding from the current study is particularly significant as it demonstrates that having collegial support influences NPs *decision latitude* and, consequently, their clinical autonomy.

The present study reveals that NPs perceived increased *decision latitude* when supported by their colleagues but not supervisors. This finding contradicts previous JCQ research in the general nursing population, where managerial and *supervisory support* was often considered more crucial, leading to increased job satisfaction (Amin et al. 2015; Baba et al. 2013; Bagheri et al. 2017, 2021; Gao et al. 2017; Orgambidez and Almeida 2020; Sasaki et al. 2020). The lack of significance of *supervisory support* for NPs might suggest that NPs have a sense of professional equality, perceiving physicians and other health professionals as their colleagues.

Dissatisfaction among nurses was associated with higher job turnover rates, which negatively impacted the health workforce resources in healthcare organisations and posed challenges to delivering safe care (Poghosyan et al. 2022). Improving satisfaction in the health workforce may be associated with continuity of care and, consequently, improved quality of care and patient outcomes. As highlighted by Sabety et al. (2021), the high turnover of healthcare clinicians disrupts patient‐care relationships, potentially diminishing health outcomes and increasing the use of urgent and emergency care services. Dery et al. (2022) emphasised the significant influence of organisational support on various aspects of nurses' practice. This support correlated with nurses working at the top of their scope of practice, having control over their tasks, and the ability to make decisions. A large cross‐sectional study of NPs by Poghosyan et al. (2022) highlighted that organisational leaders should focus on creating better work environments associated with higher job satisfaction and retention. Better work environments were defined as improving support and resources for NP roles and involving them in organisational decisions. Furthermore, the perceived support from managers was found to positively impact nurses' commitment to the organisation and effectively reduce nurse turnover (Garrosa et al. 2011; Gutierrez et al. 2012). Despite these findings related to the importance of supervisory/managerial support, the present study revealed that *supervisory support* did not significantly relate to *decision latitude*, while collegial support improved NPs' ability to make decisions about practice.

This study has identified a positive relationship between collegial support and freedom to make decisions, which improved NPs perception of their relationship with healthcare consumers. This novel finding within the NZ context highlights the significance of collegial support in enhancing NPs clinical autonomy and decision‐making, which, in turn, positively affects their relationship with healthcare consumers. Patient satisfaction has been linked to various factors in the nurses' work environment, including the care delivery model, the practice size and nurses' autonomy. Factors such as supportive professional relationships and autonomy contribute to nurses' job satisfaction and, in turn, impact patient satisfaction (Copanitsanou et al. 2017).

Interestingly, our findings suggested no relationship between the geographic location of practice and the freedom to make decisions. It may have been expected that NPs in rural settings exercised greater autonomy and scope of practice (Bae 2016; Spetz et al. 2017). However, our findings suggest that when NPs perceive themselves as valued and appreciated (*self‐identity through work*), they have more job security. Importantly, this relationship was conditional to the NPs practising in rural areas in NZ. Job insecurity, as described by Kalil et al. (2010), refers to a lack of resources and control (*decision latitude*) at work and a lack of work commitment (*self‐identity through work*). In our study, *job insecurity* was higher among metropolitan NPs than in rural regions, and the rural primary healthcare setting offered NPs greater clinical autonomy, especially in isolated areas. These findings align with the conclusions of Spetz et al. (2017), who reported that US NPs practising in rural areas had higher job satisfaction, increased clinical autonomy and a greater intent to remain in their jobs as they were able to work to the fullest extent of their scope of practice.

### 5.2 Limitations of the Study

The JCQ relies on self‐reported measures, making it susceptible to respondent bias. To address this, the questionnaire was designed with clear and straightforward language, avoiding complex structures like embedded clauses and ambiguous terms to minimise participant confusion. Although the data fit the model well, a larger sample would have been desirable to confirm construct validity in the population of New Zealand's NPs. The expected number of cases for each variable was 10 cases per 25 variables; therefore, the ideal sample size for CFA should have been at least 300 participants. The sample size of the metropolitan versus rural groups was a limitation as there were 134 participants in the metropolitan group and only 35 in the rural group. The rural population in NZ is estimated at 13.2% of the total population (Statistics New Zealand 2022).

### 5.3 Recommendations for Further Research

Further studies can explore the impact of NP‐led practice on the health outcomes of rural, remote and marginalised communities. While limited NZ studies suggest that NPs can improve access to care and health outcomes in these areas (Adams and Carryer 2019; Carryer et al. 2011), more research is needed to determine the extent to which these benefits have been realised and to identify the specific factors that contribute to their success.

### 5.4 Implications for Policy and Practice

Like many industrialised Western countries, NZ faces significant challenges in delivering appropriate healthcare services, given the high prevalence of long‐term conditions, shortage of doctors and health access inequities (Adams and Carryer 2019). In community settings, NPs with distinct professional characteristics are vital in addressing these challenges. Their attributes include collaboration, service coordination, patient education, counselling and advocacy by empowering patients and facilitating their active involvement in managing known conditions during illness and rehabilitation. NPs make a difference in the care provided, diverging from the traditional biomedical model. Grant et al. (2017) highlighted how these unique professional characteristics set NPs apart and position them as essential healthcare providers. They work with vulnerable populations, including individuals from diverse ethnicities, cultural backgrounds, socioeconomic disadvantages, remote locations, and different age and gender groups. Consequently, NPs can be a crucial link between tertiary and community primary healthcare services, ensuring continuity and comprehensive care for these populations. NPs in NZ can be part of the solution to the health system challenges, especially in primary healthcare, residential aged care settings, and remote and rural communities.

The findings of this study can inform future workforce planning in NZ. In 2019, the *Health and Disability System Review* highlighted the lack of structured planning had been a significant flaw in the current health system. The review recommended changes for the future workforce, emphasising the need to embrace interprofessional practice and enhance cultural diversity (Health and Disability System Review 2020). This study's findings revealed that NPs strongly value collegial support, pivotal in their decision‐making. This finding holds significant importance as it suggests NPs can achieve clinical autonomy. Efforts to incorporate the NP into the healthcare team would enhance collegiality and *decision latitude*. Consequently, it potentially improves healthcare outcomes and enhances New Zealanders' access to healthcare services.

## 6 Conclusion

This study has yielded invaluable findings about the psychosocial work environment of NPs by establishing the relationships between constructs of *decision latitude, social support, self‐identity through work* and *job insecurity*. Collegial support is the primary determinant of NPs' decision‐making freedom, crucial for enhancing clinical autonomy. Recognising these factors' interconnectedness is imperative to fully harness the NP workforce's potential in NZ.

## Author Contributions

I.W., A.V. substantial contributions to conception and design, or acquisition of data, or analysis and interpretation of data. A.V., E.M. involved in drafting the manuscript or revising it critically for important intellectual content. I.W., A.V., E.M. given final approval of the version to be published. Each author should have participated sufficiently in the work to take public responsibility for appropriate portions of the content. I.W., A.V., E.M. agreed to be accountable for all aspects of the work in ensuring that questions related to the accuracy or integrity of any part of the work are appropriately investigated and resolved.

## Ethics Statement

Approval for this research was sought and obtained from the Auckland University of Technology Ethics Committee (AUTEC# 20/194) on 31st July 2020.

## Conflicts of Interest

The authors declare no conflicts of interest.

## Data Availability

The data that support the findings of this study are openly available in Open Science Framework at 10.17605/OSF.IO/VT9ZP
